# Protective effect of limonin against doxorubicin-induced cardiotoxicity via activating nuclear factor - like 2 and Sirtuin 2 signaling pathways

**DOI:** 10.1080/21655979.2021.1985299

**Published:** 2021-10-08

**Authors:** Jie Deng, Min Huang, Hao Wu

**Affiliations:** a Department of Oncology, The First Affiliated Hospital of Nanjing Medical University, Nanjing, Jiangsu Province, PR China; b Department of Geriatrics, The First Affiliated Hospital of Nanjing Medical University, Nanjing, Jiangsu Province, PR China

**Keywords:** Limonin, doxorubicin, cardiotoxicity, Sirt2, Nrf2

## Abstract

The anti-tumor and anti-inflammatory effects of limonin have been established, here, we aim to explore whether limonin can induce protective effects against doxorubicin (DOX)-mediated cardiotoxicity which limits its clinical application. We found that limonin attenuated DOX-mediated cytoxicology of myocardial cell line H9C2 by measuring cell viability and reactive oxygen species (ROS) level. Additionally, limonin ameliorates DOX-induced cardiac injury in rat by examining the activity of lactate dehydrogenase (LDH), superoxide dismutase (SOD), glutathione (GSH), glutathione peroxidase (GSH-Px) and malondialdehyde (MDA) concentration, and histopathological changes. Mechanistically, it was shown that limonin partially abrogated the inhibition of Nuclear factor – like 2 and Sirtuin 2 signaling induced by DOX. Furthermore, limonin-mediated protective effects on DOX-mediated cytoxicology of H9C2 were rescued by a Sirt2-specific inhibitor or siRNA against Sirt2. Thus, this work reveals that limonin can suppress DOX-mediated cardiotoxicity by activating Nrf2 and Sirt2 signaling.

## Introduction

Doxorubicin (DOX) is a highly effective anthracycline chemotherapeutic drug, but its clinical application is limited due to its dose-dependent cardiotoxicity. However, the sensitive predictive indicators and concrete mechanisms underlying DOX-mediated cardiotoxicity are still confusing and effective therapeutic drugs are urgently needed.

Increasing evidences have shown that the monomers or extracts from Chinese herbal medicine not only have a low toxicity, but also have significant anti-tumor effects, which have become a hot spot in the research of anti-cancer drugs. In 2017, European scientists found that citrus fruit can induce preventive effects on digestive system and upper respiratory tract cancer. With the development of comprehensive utilization of citrus, various effective components have been gradually extracted and separated, especially limonin in orange peel and pomelo peel. Limonin has been shown to exert inhibitory effects on cancer progression, such as limonin can inhibit lung cancer cell proliferation [1] and intestinal carcinogenesis [2]. Specifically, limonin was recently been confirmed to attenuate the stemness of various cancer cells [3–6]. Additionally, thanks to its low toxicity and mild adverse reactions, it has attracted a growing number of attention and favor of scholars on other diseases, such as Mahmoud et al. indicate that limonin can attenuate the hepatocellular injury induced by live ischemia and reperfusion [7]; Zhang et al. reveal that limonin ameliorates acetaminophen-induced hepatotoxicity [8]; and limonin also can attenuate dextran sulfate sodium-induced chronic colitis [9]. However, its effects on DOX-mediated cardiotoxicity and related signaling pathways are never been reported.

In the present work, we first show that limonin could induce protective effects on DOX-mediated cardiotoxicity through *in vitro* and *in vivo* model. Mechanistic studies revealed that limonin rescued the inhibition of Nuclear factor – like 2 (Nrf2) and Sirtuin 2 (Sirt2) signaling induced by DOX, which is also essential for limonin-mediated protective effects.

## Material and methods

### Cell culture and reagents

Myocardial cell line H9C2 was purchased from Fenghui Biotechnology Co., Ltd (Changsha, China) and cultured in DMEM medium (Cat # PWL037, MeilunBio, Dalian, China) plus 10% FBS (Fetal bovine serum, Oricell, Suzhou, China) as well as 1% penicillin (Cat # A100339-0025, Sangon, Shanghai, China) and streptomycin (Cat # A100408-0025, Sangon). Cells were cultured at 37°C in a humidified atmosphere of 5% CO_2_. DOX was purchased from Sigma-Aldrich (Cat # 25,316–40-9, St. Louis, MO, USA). Sirt2-specific inhibitor (Cat # ab142073, AGK2) was purchased from Abcam (Cambridge, MA, USA). Sirt2 Activity Assay Kit was purchased from Abcam (cat # 156,066) and Sirt2 activity was detected following the protocols.

### Animal model

Sprague-Dawley rats (200–220 g) were purchased from GemPharmatech (Nanjing, China). Rats were housed in a standard experimental room (12 h light/dark cycle) and left for 1 week to acclimatize before starting the experiment. This study was followed by the Guide for the Care and Use of Laboratory Animals (NIH Publication No. 85–23, Revised 2016). All experimental procedures were following with the guidelines of the research ethics committee of Nanjing Medical University (Nanjing, China). Here, rats were randomly separated into four groups: control (0.9% saline), DOX treatment (10 mg/kg), and DOX plus Limonin treatment (5 and 10 mg/kg, Cat # 1180–71-8, Solarbio, Beijing, China). For control groups, it was administered the vehicles (0.9% saline) of the used drugs (every other day); For the DOX groups, they were received the vehicle (0.9% saline) of limonin for 1 week (every other day) and i.p. injection of DOX (10 mg/kg) on the fifth day of the experiment (every other day for four injections) without limonin injection; For limonin group, it was administered limonin (10 mg/kg) (every other day); For DOX and limonin treatment groups, they were received a daily dose of limonin (5 mg/kg i.p or 10 mg/kg i.p) for 1 week and i.p. injection of DOX (10 mg/kg) on the fifth day of the experiment (every other day for four injections) without limonin injection. Seven days after the DOX last administration, the rats were euthanized and subjected to other experiments.

### Sample collection

By the seventh day of the experiment, rats were anesthetized with urethane (20% in a dose of 1 g/kg) i.p. Blood was withdrawn from the abdominal aorta, and then centrifuged at 5000 rpm for 15 min (JanetzkiT30 centrifuge, Germany) for separation of clear sera to be used in the detection of cardiac enzymes. Hearts were dissected, washed with normal saline. Part of the heart tissue was homogenized in 5% w/v ice-cold phosphate-buffered saline (PBS) (0.01 M, pH 7.4); then, homogenate was centrifuged at 3000 rpm for 20 min and the supernatant was stored at −80°C till used for biochemical analysis. Another part of heart tissue was stored in −80°C for western blotting detection of proteins’ level. Lastly, samples of cardiac tissue were stored in 10% formalin for histopathological and immunohistochemical study.

### Histopathological examinations

The heart was fixed with 4% paraformaldehyde, embedded in paraffin and dehydrated in a series of ethanol. Then, tissue samples were cut into 5 μm slicers which were used for staining. Briefly, paraffin sections were dewaxed and hydrated followed by dying with hematoxylin and eosin (HE) solution for 20 mins and washed with tap water. Then, sections were differentiated with differentiation medium. Subsequently, sections were soaked in warm water (about 50°C) for 5 mins and placed into eosin dye solution for 2 mins, and rinsed with tap water. Sections were soaked in tap water for another 5 mins, dehydrated, transparent and sealed. Finally, the stained slicers were photographed under a microscope (VHX – 7000, Keyence, Osaka, Japan). The cardiac injury was classified on the degree of vascular congestion, hemosiderosis, cloudy fatty degeneration, loss of muscle striation, apoptosis, and necrosis. The severity of the pathological lesions was graded semi-quantitatively depending on the degree and extent of the alteration as follows: score 0 was considered normal, score 1 (+) for mild changes, score 2 (++) for moderate changes, and score 3 (+++) for severe changes.

### TUNEL staining assay

Cardiomyocyte apoptosis was analyzed by TUNEL staining using In Situ Cell Death Detection Kit (Roche Diagnostics GmbH, Mannheim, Germany). Briefly, after being fixed with 4% paraformaldehyde, CMs were incubated with permeabilizing solution for 30 min and were then treated in TUNEL reaction mixture for 1 h at 37°C. Morphological assessment was performed by fluorescence microscopy (20 × objective). Nine microscopic fields were randomly selected to observe at least 100 cells to assess apoptosis.

### Cell viability assay

H9C2 cells were seeded into 96-well plates in a concentration of 4000 cells/well. After 12 h, different concentrations of DOX (0.1 μM, 0.5 μM, 1 μM, 5 μM, 25 μM, 125 μM) were added into medium as well as different concentrations of limonin (1 μM, 5 μM, 25 μM). 48 h later, cell viability was detected using CCK8 (Cat # HB-CCK8-1, Cell Counting Kit-8, Hanbio, Shanghai, China) method.

### Examination of reactive oxygen species (ROS)

H9C2 cells were seeded into 6-well plates in a concentration of 5 × 10^5^ cells/well. 12 h later, cells were treated with DOX (1 μM) as well as limonin (5 μM) or not. After another 36 h, ROS level was examined using ROS Assay Kit (Cat # S0033S, Beyotime, Beijing, China).

### Analysis of biochemical index

The activity of lactate dehydrogenase (LDH), creatine kinase (CK), superoxide dismutase (SOD), glutathione (GSH) and glutathione peroxidase (GSH-Px), and the concentration of malondialdehyde (MDA) were measured in rat heart tissues after being homogenized and obtaining the supertant using LDH Cytotoxicity Assay Kit (Cat # C0016, Beyotime), CK assay kits (Cat # A032-1-1, Nanjing Jiancheng Bioengineering Institute, Nanjing, China), Lipid Peroxidation MDA Assay Kit (Cat # S0131S, Beyotime), Total Superoxide Dismutase Assay Kit with WST-8 (Cat # S010S, Beyotime), GSH and GSSG Assay Kit (Cat # S0053, Beyotime) and Total Glutathione Peroxidase Assay Kit with NADPH (Cat # S0058, Beyotime), respectively.

### Western blot assay

H9C2 cells with different treatment were lysed, quantified and subjected to SDS-PAGE electrophoresis. Proteins were transferred to PVDF membranes (Cat # 1,620,177, Bio-Rad, Hercules, CA, USA) which were incubated with 5% nonfat milk in TBST and followed by incubation of primary antibodies at 4°C overnight. Then the PVDF membranes were washed with TBST and incubated with secondary antibodies. Protein expression was detected using BeyoECL Moon (Cat # P0018FS, Beyotime) under a Tanon 5200 machine (Shanghai, China). The detailed primary and secondary antibodies were listed as below: anti-cleaved caspase 3 (Cat # E83-77, Abcam); anti-caspase 3 (Cat # ab32351, Abcam); anti-Bax (Cat # WL01637, Wanleibio, Shenyang, China); anti-Bcl-2 (Cat # WL01556, Wanleibio); anti-β-actin (Cat # WL01372, Wanleibio); anti-Nrf2 (Cat # WL02135, Wanleibio); anti-Sirt2 (Cat # ab211033, Abcam); anti-HO-1 (Cat # EP1391Y, Abcam); anti-GST (Cat # ab138491, Abcam); anti-NQO1 (Cat # 11,451-1-AP, Proteintech, Wuhan, China); anti-FOXO3a (Cat # 66,428-1-Ig, Proteintech); anti-GCLM (Cat # 14,241-1-AP, Proteintech); anti-Keap1 (Cat # 10,503-2-AP, Proteintech). IgG(H + L)(HRP-labeled Goat Anti-Rabbit IgG(H + L)) and IgG(H + L)(HRP-labeled Goat Anti-Mouse IgG(H + L)) were purchased from Beyotime (Beijing, China). All protein expressions were normalized by β-actin expression.

### Small interfering RNA (siRNA) synthesis and transfection

The siRNA against Sirt2 and corresponding negative control (NC) were purchased from GenePharma (Shanghai, China). The transfection procedure was performed using Magic™ siRNA (Cat # CBMS250) according to the manufacturer’s recommendation.

### Statistical analyses

Data were denoted as the mean ± standard deviation (SD), single factor analysis of variance (ANOVA) was used for analysis, and non-paired *t* test was used for inter-group comparison. P < 0.05 was considered to be statistically significant.

## Results

### Limonin improves cell viability of H9C2 cells suppressed by DOX treatment

**Figure 1.**
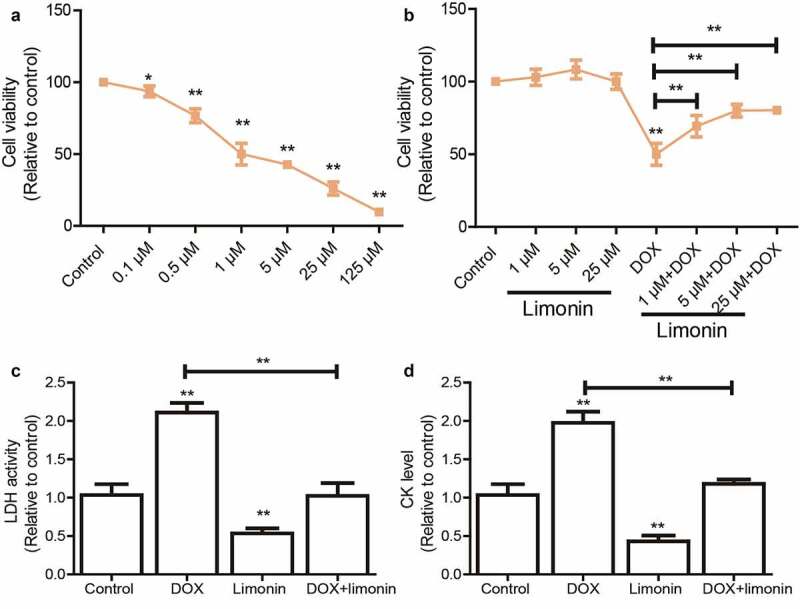
Limonin improves cell viability of H9C2 cells suppressed by DOX treatment. (a) Cell viability of H9C2 cells was evaluated after being treated with different concentrations of DOX. (b) Cell viability of H9C2 cells was determined after being treated with different concentrations of limonin or limonin plus DOX. (c) LDH activity was detected in H9C2 cells with DOX treatment as well as limonin or not. (d) CK level was measured in H9C2 cells with DOX treatment as well as limonin or not. *p < 0 05, **p < 0 01 vs control

We initially confirmed that DOX significantly reduced H9C2 cell viability in a concentration-dependent manner (Figure 1(a)). As shown in the dose-response curves, the median lethal concentration (LC_50_) was about 1 μM for the 48 h time point, which was chosen for the treatment in this work. Then, H9C2 cells were pre-treated with different concentrations of limonin before being treated with DOX, it was found that limonin pretreatment attenuated DOX-mediated inhibition on the cell viability of H9C2 cells in a dose-dependent manner (Figure 1(b)). Notably, a higher concentration of limonin more than 25 μM showed no additional protective effects on cell viability; thus, the 5 μM of limonin was selected for the subsequent experiment *in vitro*. Additionally, LDH level was markedly upregulated by DOX treatment, which was rescued by limonin treatment (Figure 1(c)). Consistently, DOX-induced upregulation of CK level was partially abrogated by limonin treatment (Figure 1(d)).

### Limonin attenuates DOX-mediated apoptosis on H9C2 cells

**Figure 2.**
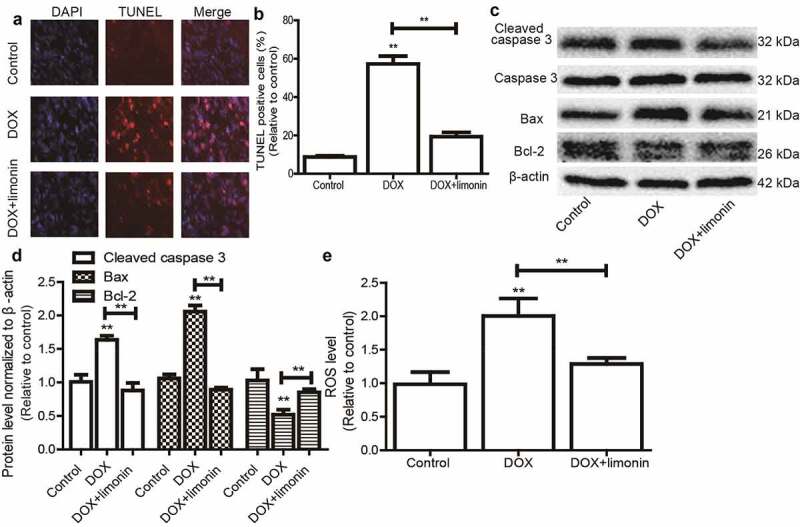
Limonin attenuates DOX-mediated apoptosis on H9C2 cells. (a and b) TUNEL-positive cells were examined in H9C2 cells with or without DOX treatment as well as limonin or not (a), and quantified (b). (c and d) The protein expression of apoptotic executors (Cleaved caspase 3, Bax, Bcl-2) was detected in H9C2 cells (c), and quantified (d) as depicted in (A). (e) ROS level was determined in H9C2 cells as depicted in (A). **p < 0 01 vs control

We continued to investigate the effects of limonin on DOX-mediated apoptosis of H9C2 cells. As shown in Figure 2(a,b), DOX remarkably induced H9C2 cell apoptosis as evident by the increase TUNEL-positive cells, this effect was attenuated by limonin treatment. Additionally, the expression of apoptotic executors (cleaved caspase3, Bax) was increased by DOX treatment, while the expression of apoptotic suppressor (Bcl-2) was decreased by DOX treatment, this effect was also rescued by limonin treatment (Figure 2(c,d)). Furthermore, DOX-induced upregulation of ROS was reduced by limonin treatment (Figure 2(e)). These results suggest that limonin can attenuate DOX-induced cytoxicology of H9C2 cells.

### Limonin ameliorates DOX-induced cardiac injury in rat

**Figure 3.**
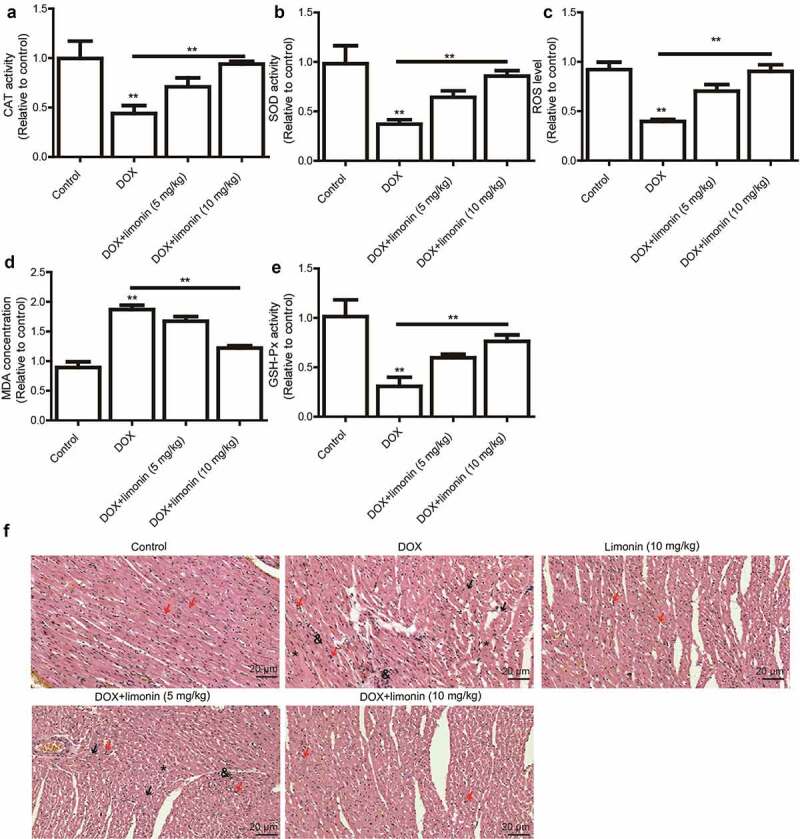
Limonin ameliorates DOX-induced cardiac injury in rat. (a) CAT activity was detected in heart tissues from rat treated differently as indicated. (b) SOD activity was examined in heart tissues from rat treated differently as indicated. (c) ROS level was determined in heart tissues from rat treated differently as indicated. (d) MDA activity was evaluated in heart tissues from rat treated differently as indicated. (e) GSH-Px activity was measured in heart tissues from rat treated differently as indicated. **p < 0 01 vs control. (f) Myocardial cell injury was detected heart tissues from rat treated differently as indicated. Control group: showing branched striated muscle fibers with central vesicular nuclei and light eosinophilic cytoplasm. Some blood capillaries present in between fibers. DOX group: showing area of degenerated wavy muscle fibers with absent striations (black arrows). Some fibers appear with pyknotic nuclei (red arrows), others with absent nuclei (*). Notice marked congested blood capillaries with inflammatory cells infiltration (&) and hemosiderin pigment (#) between muscle fibers. Limonin group: showing branched striated muscle fibers with central vesicular nuclei and light eosinophilic cytoplasm. Some blood capillaries present in between fibers. DOX + limonin (5 mg/kg): showing some areas of degenerated muscle fibers with absent striations (black arrows), while others are showing preserved muscle fiber with central oval nuclei (red arrows). Notice marked scattered dilated congested capillaries (c) with cloudy fatty degeneration and intermuscular edema (#). DOX + limonin (10 mg/kg) group: showing areas of preserved muscle fiber striations with central oval nuclei (red arrows). Notice some dilated congested capillaries (c) with minimal areas of edema between fibers. (HE× 200, scale bar = 20 µm)

As oxidative stress is regarded as the primary cause of DOX-induced cardiomyopathy, we detected the levels of oxidative stress markers (CAT, SOD, MDA, GSH, GSH-Px) in rat heart tissues. As shown in Figure 3(a,c), the cardiac activity of CAT and SOD, and ROS level were remarkably decreased by DOX treatment, which was rescued by limonin treatment. Additionally, MDA activity was increased by DOX treatment, this effect was attenuated by limonin treatment (Figure 3(d)). Furthermore, DOX-induced downregulation of GSH-Px level was partially reversed by limonin treatment (Figure 3(e)). Consistently, the heart tissues from rat exposed to DOX showed myocardial cell injury, as characterized by the interstitial edema, capillary congestion and inflammation infusion, this effect was ameliorated by limonin treatment (Figure 3(f)).

### Limonin attenuates the Nrf2 and Sirt2 signaling inactivated by DOX

**Figure 4.**
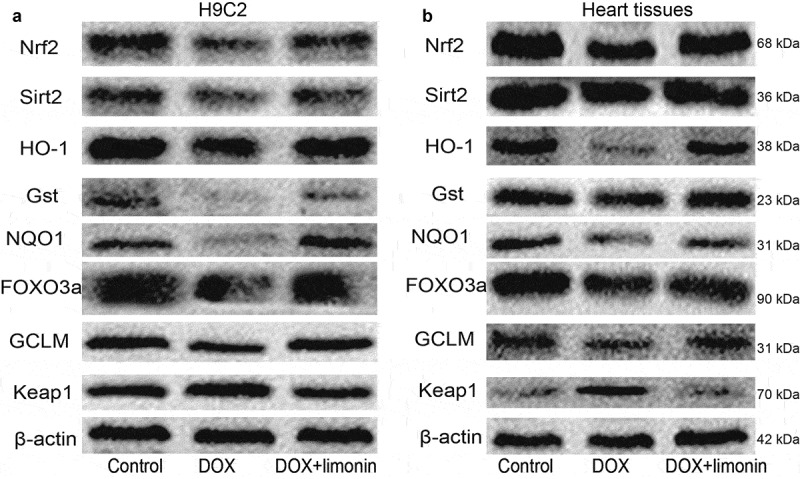
Limonin attenuates the Nrf2 and Sirt2 signaling inactivated by DOX. (a) The protein expression of Nrf2 and Sirt2, and their downstream effectors was examined in H9C2 cells with or without DOX treatment plus limonin or not. DOX: 1 μM, Limonin: 5 μM. (b) The protein expression of Nrf2 and Sirt2, and their downstream effectors was examined in heart tissues from rat treated differently as indicated. DOX: 10 mg/kg, Limonin: 10 mg/kg

We then explored the mechanisms underlying limonin-mediated protective effects on DOX-induced cardiotoxicity. As the inhibition of Nrf2 and Sirt2 signaling has been shown to be responsible for DOX-mediated cardiotoxicity, the expression levels of Nrf2, Sirt2 and some of their downstream effectors (HO-1, Gst, NQO1, FOXO3a, GCLM, Keap1) were examined. As shown in Figure 4(a), the protein expression of Nrf2, Sirt2, HO-1, Gst, NQO1, FOXO3a, GCLM was inhibited by DOX treatment, while Keap1 expression was increased by DOX treatment, this effect was rescued by limonin treatment. Consistently, western blot analysis on rat heart tissues obtained similar results (Figure 4(b)).

### Limonin ameliorates DOX-induced cardiotoxicity dependent on Sirt2

**Figure 5.**
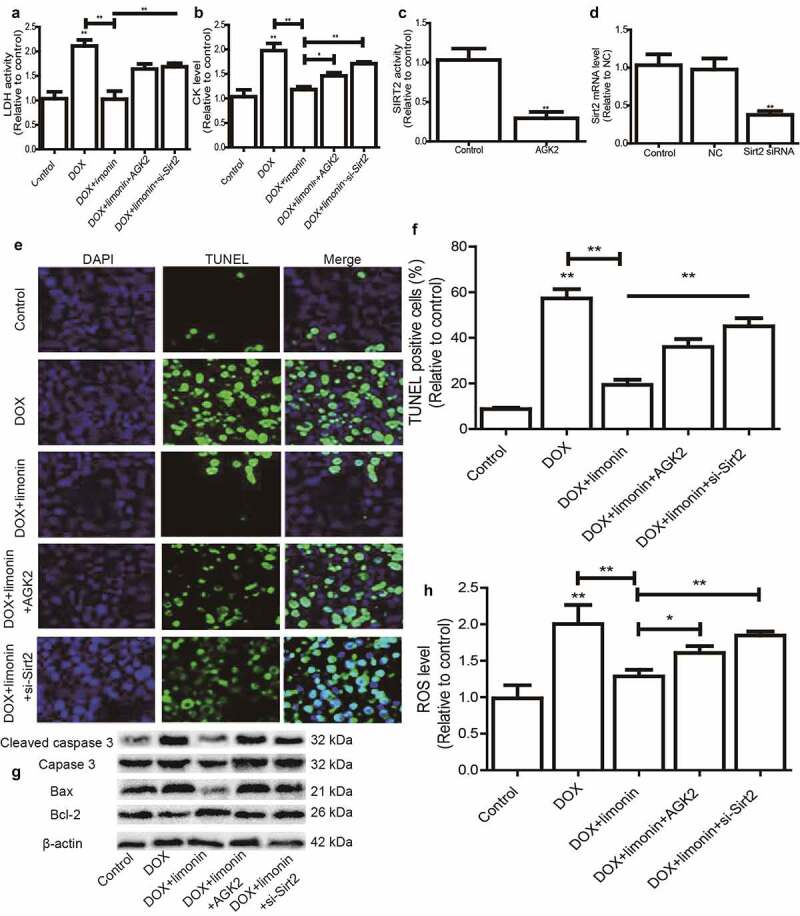
Limonin ameliorates DOX-induced cardiotoxicity dependent on Sirt2. (a) LDH activity was detected in H9C2 cells treated differently as indicated. (b) CK level was examined in H9C2 cells treated differently as indicated. (c) Sirt2 activity was measured in H9C2 cells with or without AGK2 treatment. (d) Sirt2 mRNA level was detected in H9C2 cells transfected with or without Sirt2 siRNA. (e and f) TUNEL-positive cells were determined in H9C2 cells treated differently as indicated (e) and quantified (f). (g) The protein expression of apoptotic executors was evaluated in H9C2 cells treated differently as indicated. (h) ROS level was measured in H9C2 cells treated differently as indicated. *p < 0 05, **p < 0 01 vs control. DOX: 1 μM, Limonin: 5 μM, AGK2: 1 μM, si-Sirt2: 50 nM

Finally, we investigated the essential role of Sirt2 on limonin-mediated protective effects on DOX-induced cardiotoxicity. Sirt2-specific inhibitor (AGK2) was added or Sirt2 siRNA was transfected in H9C2 cells with DOX treatment plus limonin incubation, it was found that limonin-mediated decrease of LDH and CK level in DOX-treated H9C2 cells was rescued by Sirt2 inhibition or knockdown (Figure 5(a,b)). The inhibition efficiency of AGK2 and knockdown efficiency of Sirt2 siRNA were confirmed in H9C2 cells, respectively (Figure 5(c,d)). Additionally, the protective effects of limonin on DOX-induced oxidative injury on H9C2 cells were partially abrogated by Sirt2 inhibition or knockdown, as evident by the change of TUNEL-positive cells (Figure 5(e,f)), apoptotic regulator expression (Figure 5(g)) and ROS level (Figure 5(h)). Therefore, these results indicate that SIRT2 is necessary for limonin-mediated protective effects on DOX-induced cardiotoxicology.

## Discussion

The cardiotoxicity of DOX has become the major obstacle for its clinical application; however, the underlying mechanisms contributing to DOX-mediated cardiotoxicity are still confusing. Thus, it is very important to elucidate the mechanisms, based on which potential drugs can be explored. Here, we identified that limonin can ameliorate DOX-mediated cardiotoxicity through *in vitro* and *in vivo* experiments. This is the first study, to our knowledge, revealing the limonin-mediated protective effects on DOX-mediated cardiotoxicity.

An increasing number of evidences have explored potential protective agents based on chemical drugs; however, the toxic dose and effective dose of protective agents based on the research of chemical drugs are similar, and the side effects of are aggravated when they are combined with chemotherapeutic drugs [10,11], which cannot be widely used in clinic. Recently, a large number of studies have found potential drugs that can improve the cardiotoxicity of DOX from the resources of traditional Chinese medicine, such as Zaid H Maayah et al. showed that resveratrol reduced DOX-induced cardiotoxicity in juvenile rat through inactivating the NLRP3-inflammasome [12]; Wenna Zhou et al. indicated that two alkaloids from hippophae rhamnoides linn. inhibited DOX-induced apoptosis of cardiac muscle cells via suppressing JNK signaling pathway [13]; Samar S Elblehi et al. demonstrated that date palm pollen extract averts DOX-induced cardiomyopathy fibrosis by suppressing apoptosis-targeting Bax/Bcl-2 and caspase-3 signaling pathways [14]. Limonin and other analogues are the main reasons for the bitterness of citrus fruits, which are generally enriched in citrus fruits, especially in seeds. Limonin has been shown to hold a wide range of biological activities, such as anti-tumor, anti-virus, anti-inflammatory and so on. However, its roles in protecting DOX-induced cardiotoxicity have never been investigated.

There are several mechanisms contributing to DOX-induced cardiotoxicity, for example, NLRP3-inflammasome activation [12,15] and cell death pathways including autophagy, ferroptosis, necroptosis, pyroptosis and apoptosis [16]. Here, we found that Nrf2 and Sirt2 signaling pathways were inhibited by limonin *in vitro* and *in vivo* by examining the expression levels of Nrf2, Sirt2 and some of their downstream effectors (HO-1, Gst, NQO1, FOXO3a, GCLM, Keap1). Importantly, Nrf2 and Sirt2 signaling pathways have been confirmed to be critically involved in DOX-induced cardiotoxicity [17,18]. Our results further strengthened these two proteins, Nrf2 and Sirt2, could be potential targets for DOX-induced cardiotoxicity. In addition, the previous study has shown that miR-140-5p could aggravate DOX-induced cardiotoxicity via targeting both Nrf2 and Sirt2 [17]. Plenty of evidences have shown that miRNAs are engaged in DOX-induced cardiotoxicity, such miR-133b [19], miR-98 [20] and miR-152 [21]. However, it is still unclear whether miR-140-5p is involved in limonin-mediated protection on DOX-induced cardiotoxicity, which could be explored in the future.

## Conclusions

Taken together, this study first revealed the protective effects of limonin on DOX-induced cardiotoxicity. Although more experiments are needed to reveal its concrete mechanisms underlying limonin-mediated effects, limonin might be a penitential drug for DOX-induced cardiotoxicity.

## Limitation/short-coming of the study

The concrete targets of limonin-mediated protective effects on DOX-induced cardiotoxicity are still unclear.

## Future direction

Further transcriptome-sequencing and Co-IP combined with LC-MS technology should be constructed to reveal the concrete targets of limonin responsible for DOX-induced cardiotoxicity.

## Data Availability

All data generated or analyzed during this study are included in this published article.

## References

[1] Gong C, Qi L, Huo Y, et al. Anticancer effect of Limonin against benzo(a)pyrene-induced lung carcinogenesis in Swiss aslbino rat and the inhibition of A549 cell proliferation through apoptotic pathway. J Biochem Mol Toxicol. 2019;33(12):e22374. 31702096 10.1002/jbt.22374

[2] Shimizu S, Miyamoto S, Fujii G, et al. Suppression of intestinal carcinogenesis in *Apc*-mutant mice by limonin. J Clin Biochem Nutr. 2015;57(1):39–43. 26236099 10.3164/jcbn.15-28 PMC4512898

[3] Zhao W, Wu M, Cui L, et al. Limonin attenuates the stemness of cervical carcinoma cells by promoting YAP nuclear-cytoplasmic translocation. Food Chem Toxicol. 2019;125:621–628. 30738134 10.1016/j.fct.2019.02.011

[4] Tang Z, Tang Y, Li L, et al. Limonin provokes hepatocellular carcinoma cells with stemness entry into cycle via activating PI3K/Akt signaling. Biomed Pharmacother. 2019;117:109051. 31177062 10.1016/j.biopha.2019.109051

[5] Su Z, Wang C, Chang D, et al. Limonin attenuates the stemness of breast cancer cells via suppressing MIR216A methylation. Biomed Pharmacother. 2019;112:108699. 30970511 10.1016/j.biopha.2019.108699

[6] Gao L, Sang JZ, Cao H. Limonin enhances the radiosensitivity of nasopharyngeal carcinoma cells via attenuating Stat3-induced cell stemness. Biomed Pharmacother. 2019;118:109366. 31545261 10.1016/j.biopha.2019.109366

[7] Mahmoud MF, Gamal S, El-Fayoumi HM. Limonin attenuates hepatocellular injury following liver ischemia and reperfusion in rats via toll-like receptor dependent pathway. Eur J Pharmacol. 2014;740:676–682. 24967531 10.1016/j.ejphar.2014.06.010

[8] Yang R, Song C, Chen J, et al. Limonin ameliorates Acetaminophen-induced hepatotoxicity by activating Nrf2 antioxidative pathway and inhibiting NF-κB inflammatory response via upregulating Sirt1. Phytomedicine. 2020;69:153211. 32259676 10.1016/j.phymed.2020.153211

[9] Song C, Chen J, Li X, et al. Limonin ameliorates dextran sulfate sodium-induced chronic colitis in rat by inhibiting PERK-ATF4-CHOP pathway of ER stress and NF-κB signaling. Int Immunopharmacol. 2021;90:107161. 33168409 10.1016/j.intimp.2020.107161

[10] Gandhi L, Harding MW, Neubauer M, et al. A phase II study of the safety and efficacy of the multidrug resistance inhibitor VX-710 combined with doxorubicin and vincristine in patients with recurrent small cell lung cancer. Cancer. 2007;109(5):924–932. 17285598 10.1002/cncr.22492

[11] Minderman H, O’Loughlin KL, Pendyala L, et al. VX-710 (biricodar) increases drug retention and enhances chemosensitivity in resistant cells overexpressing P-glycoprotein, multidrug resistance protein, and breast cancer resistance protein. Clin Cancer Res. 2004;10(5):1826–1834. 15014037 10.1158/1078-0432.ccr-0914-3

[12] Maayah ZH, Alam AS, Takahara S, et al. Resveratrol reduces cardiac NLRP3-inflammasome activation and systemic inflammation to lessen doxorubicin-induced cardiotoxicity in juvenile rat. Molecules. 2021. 10.1002/1873-3468.14091 PMC8608383 33876420

[13] Zhou W, Ouyang J, Hu N, et al. Protective effect of two alkaloids from hippophae rhamnoides linn. against doxorubicin-Induced toxicity in H9c2 cardiomyoblasts. Animals (Basel). 2021;26. 10.3390/molecules26071946 PMC8037594 33808398

[14] Elblehi SS, El-Sayed YS, Soliman MM, et al. Date palm pollen extract avert doxorubicin-Induced cardiomyopathy fibrosis and associated oxidative/nitrosative stress, inflammatory cascade, and apoptosis-Targeting Bax/Bcl-2 and caspase-3 signaling pathways. FEBS Lett. 2021;11. 10.3390/ani11030886 PMC8003775 33804672

[15] Liu J, Jin Y, Wang B, et al. Dopamine D1 receptor alleviates doxorubicin-induced cardiac injury by inhibiting NLRP3 inflammasome. Biochem Biophys Res Commun. 2021;561:7–13. 33992835 10.1016/j.bbrc.2021.04.098

[16] Christidi E, Brunham LR. Regulated cell death pathways in doxorubicin-induced cardiotoxicity. Cell Death Dis. 2021;12(4):339. 33795647 10.1038/s41419-021-03614-x PMC8017015

[17] Zhao L, Qi Y, Xu L, et al. MicroRNA-140-5p aggravates doxorubicin-induced cardiotoxicity by promoting myocardial oxidative stress via targeting Nrf2 and Sirt2. Redox Biol. 2018;15:284–296. 29304479 10.1016/j.redox.2017.12.013 PMC5975069

[18] Zhao D, Xue C, Li J, et al. Adiponectin agonist ADP355 ameliorates doxorubicin-induced cardiotoxicity by decreasing cardiomyocyte apoptosis and oxidative stress. Biochem Biophys Res Commun. 2020;533(3):304–312. 32958254 10.1016/j.bbrc.2020.09.035

[19] Li Z, Ye Z, Ma J, et al. MicroRNA‑133b alleviates doxorubicin‑induced cardiomyocyte apoptosis and cardiac fibrosis by targeting PTBP1 and TAGLN2. Int J Mol Med. 2021;48(1). DOI:10.3892/ijmm.2021.4958 PMC8128419 33982775

[20] Pan Y, Pan YM, Liu FT, et al. MicroRNA-98 ameliorates doxorubicin-induced cardiotoxicity via regulating caspase-8 dependent Fas/RIP3 pathway. Environ Toxicol Pharmacol. 2021;85:103624. 33617954 10.1016/j.etap.2021.103624

[21] Zhang WB, Lai X, Guo XF. Activation of Nrf2 by miR-152 inhibits doxorubicin-induced cardiotoxicity via attenuation of oxidative stress, inflammation, and apoptosis. Oxid Med Cell Longev. 2021;2021:8860883. 33574984 10.1155/2021/8860883 PMC7857911

